# Complicated Growth Plate Fracture: Torn Metaphyseal Periosteum with Physeal Interposition

**DOI:** 10.5334/jbsr.2644

**Published:** 2021-12-10

**Authors:** Frederiek Laloo, Saira Haque, Marcela De La Hoz Polo

**Affiliations:** 1 Ghent University Hospital, BE; 2 King’s College Hospital, London, GB

**Keywords:** Magnetic Resonance Imaging, Pediatrics, Growth plate, Salter-Harris Fractures, Periosteal physeal interposition

## Abstract

**Teaching point:** Displacement of torn periosteum into the growth plate is an uncommon pediatric entity following trauma – which can be seen on an MRI as low signal intensity physeal interposition on all pulse sequences, and requires open surgical reduction, as it may lead to growth plate bridging and subsequent extremity length discrepancy.

## Case

A 12-year-old boy was referred to the accident and emergency department. He had sustained an acute left knee injury with severe pain and was unable to weight-bear. No previous medical history was known.

**Figure 1:**
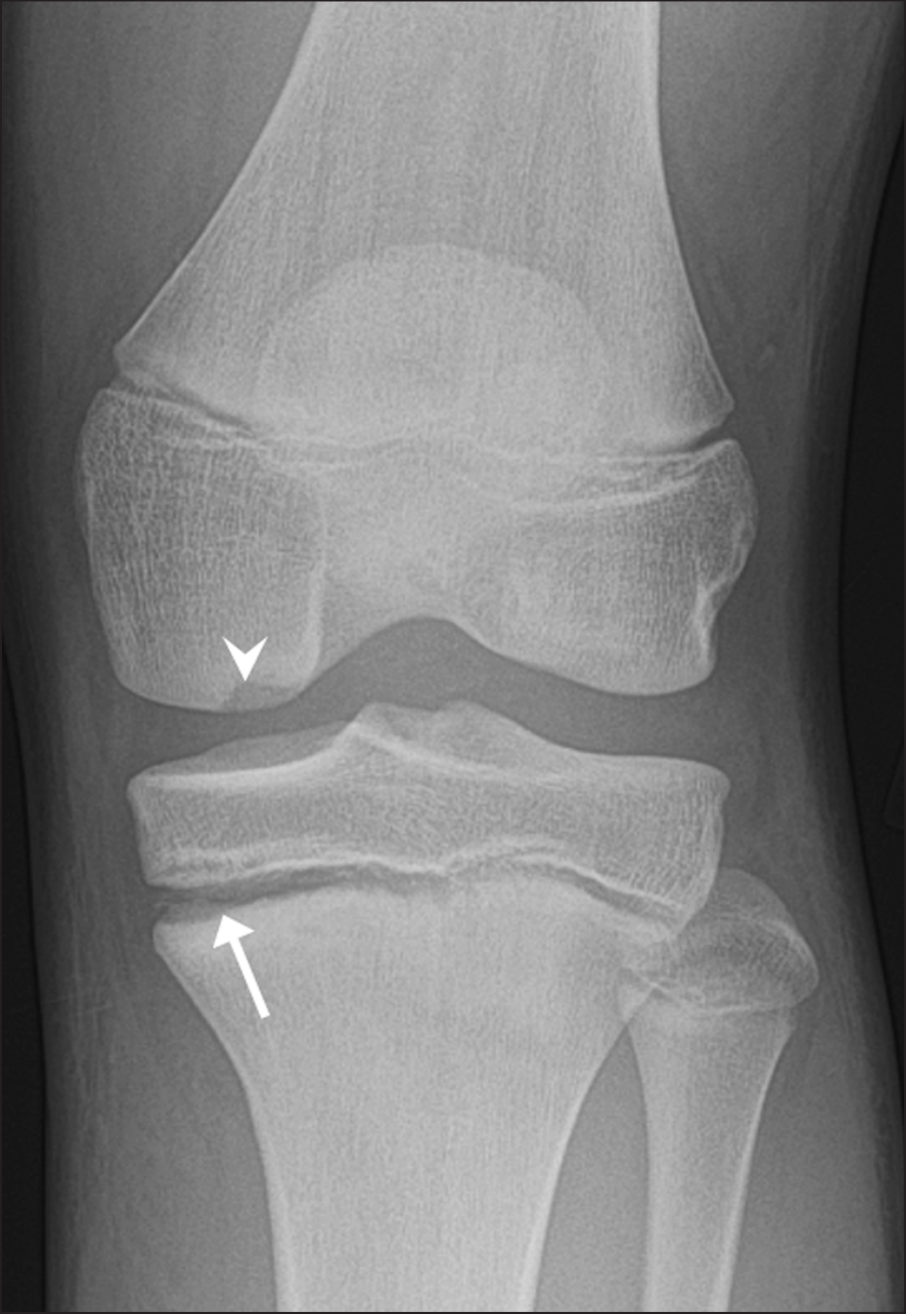


Frontal radiography of the left knee demonstrated two abnormalities: an osteochondral defect at the medial femur condyle (***Figure 1**, arrowhead*) and widening of the proximal medial tibial growth plate (***Figure 1**, arrow*), in keeping with a Salter-Harris type I fracture. Also note the soft tissue swelling along the medial side of the knee.

**Figure 2:**
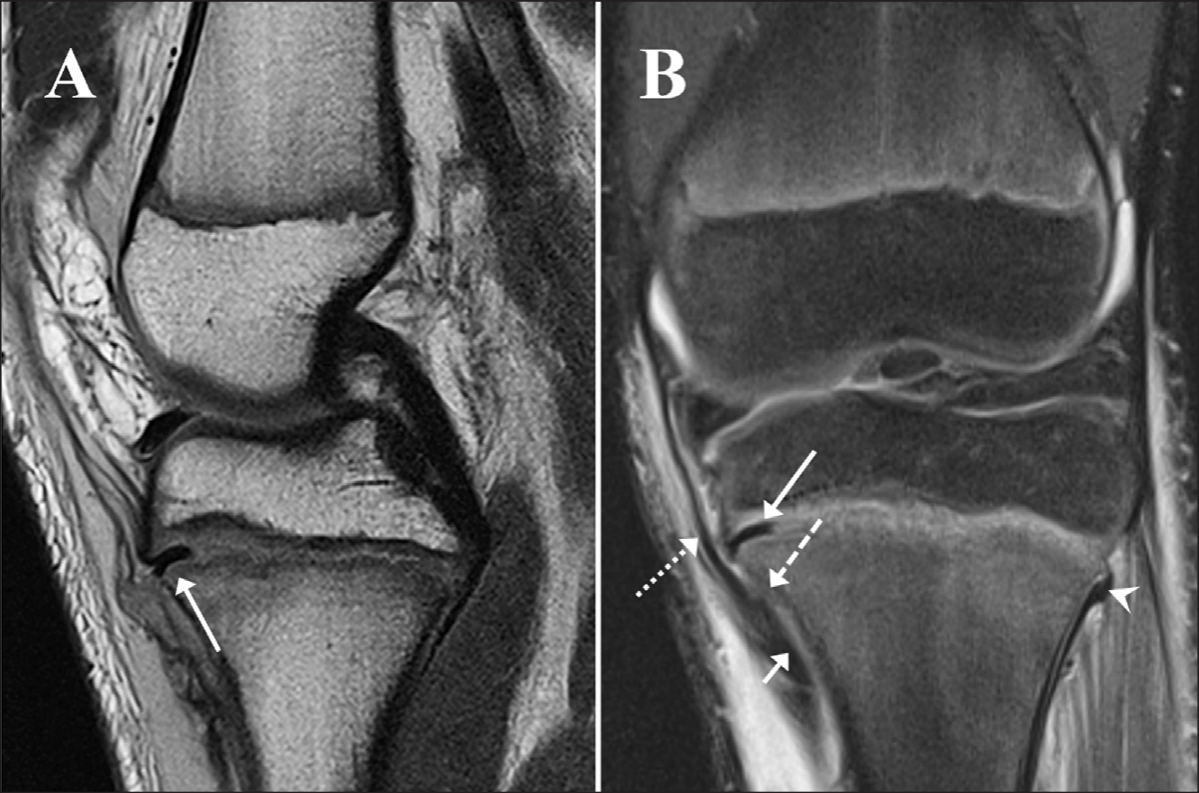


MRI of the left knee was obtained because of persistent widening of the proximal tibia physis. Proton density and fat suppressed proton density sequences confirmed the Salter-Harris I fracture of the medial proximal tibial physis with mild widening and a well-defined band of low signal intensity on all sequences in the medial part of the growth plate (***Figure 2A** and **2B**, long arrow*) representing trapped periosteum that has displaced from the medial tibial metaphysis (*dashed arrow*). Also note the slight periosteal stripping and tear along the lateral tibial metaphysis without displacement (***Figure 2B**, arrowhead*). Additionally, there was a moderate grade injury of the medial collateral ligament and pes anserine, which are partially shown (***Figure 2B**, dotted arrow and short arrow*), and extensive soft tissue oedema.

The osteochondral lesion at the medial femoral condyle had normal overlying cartilage without signs of instability (not depicted).

## Comment

Distraction injuries can cause disruption of the epiphyseal-metaphyseal unit, causing stripping of the periosteum from the underlying bony cortex on the distraction side. The torn periosteum can subsequently herniate into the physis, as in this case.

Interposition of periosteum into the growth plate following trauma is rare. It is however important to recognize early on – as the interposed periosteum will cause bony bridging and growth arrest, which can lead to discrepancy in length of the extremities. Besides periosteum, other soft tissues structures may also be the cause of physeal interposition, such as ligament, tendon, muscle, or neurovascular bundle. Interposition of soft tissue in the growth plate should be suspected when post-reduction radiographs show persistent growth plate widening of more than 3 mm. If confirmed, open surgical reduction will be required [1].

## References

[1] Chen J, Abel MF, Fox MG. Imaging appearance of entrapped periosteum within a distal femoral Salter-Harris II fracture. Skeletal Radiol. 2015; 44: 1547–1551. DOI: 10.1007/s00256-015-2201-x 26138340

